# Correction: Tong et al. Reduced Apoptotic Injury by Phenothiazine in Ischemic Stroke Through the NOX-Akt/PKC Pathway. *Brain Sci.* 2019, *9*, 378

**DOI:** 10.3390/brainsci16030331

**Published:** 2026-03-20

**Authors:** Yanna Tong, Kenneth B. Elkin, Changya Peng, Jiamei Shen, Fengwu Li, Longfei Guan, Yu Ji, Wenjing Wei, Xiaokun Geng, Yuchuan Ding

**Affiliations:** 1 Luhe Institute of Neuroscience, Capital Medical University, Beijing 101100, China; 2 Department of Neurology, Luhe Clinical Institute, Capital Medical University, Beijing 101100, China; 3 Department of Neurosurgery, Wayne State University School of Medicine, Detroit, MI 48201, USA; 4 Department of Research and Development Center, John D. Dingell VA Medical Center, Detroit, MI 48201, USA; 5 Department of General Surgery, Luhe Clinical Institute, Capital Medical University, Beijing 101100, China; 6 China-America Institute of Neuroscience, Xuanwu Clinical Institute, Capital Medical University, Beijing 100053, China

In our article “Reduced Apoptotic Injury by Phenothiazine in Ischemic Stroke Through the NOX-Akt/PKC Pathway” [1], we identified an error in Figure 1A introduced during figure preparation. The TTC images for the no-treatment, chlorpromazine and promethazine (C + P), and C + P/NADPH oxidase (NOX) inhibitor groups were incorrectly presented. The corrected Figure 1 is provided below. This error does not affect the results or conclusions of the study.

The authors state that the scientific conclusions are unaffected. This correction was approved by the Academic Editor. The original publication has also been updated.

**Figure 1:**
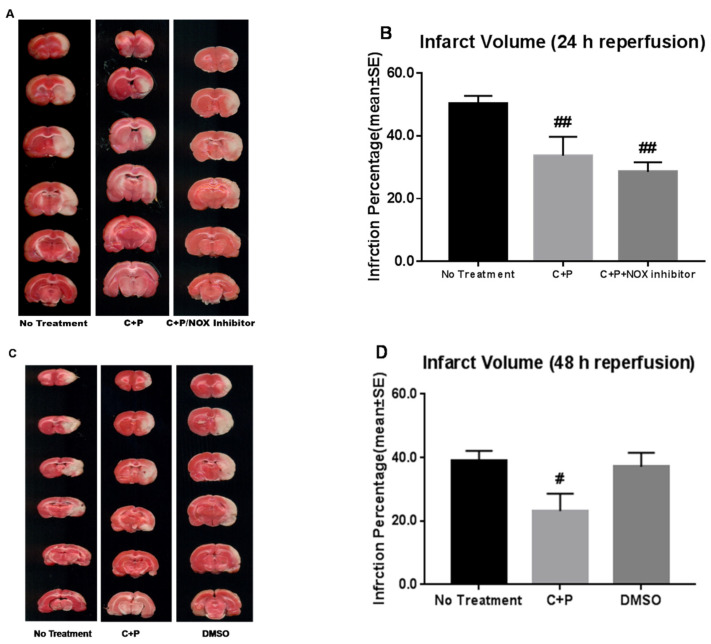
Infarct volume reduction by control treatment, chlorpromazine and promethazine (C+P) treatment, and C+P/NADPH oxidase (NOX) inhibitor treatment. 2,3,5-Triphenyltetrazolium chloride (TTC) histology depicts (**A**) the cortex and striatum at three different levels supplied by the middle cerebral artery (MCA) from anterior +1.00 mm to posterior −4.8 mm to the bregma at 24 h of reperfusion. (**B**) Percentage of infarct volume reduction (mean ± standard error (SE)) with no treatment (50.0% ± 2.5%), C+P treatment (33.7% ± 6.0%), and C+P/NOX inhibitor treatment (28.5% ± 3.0%) at 24 h of reperfusion. While no significant difference in infarct volume was produced between cohorts, there was a significant reduction in both cohorts when compared to no treatment (## *p* < 0.01). In addition, at 48 h of reperfusion (**C**,**D**), infarct volume in ischemic rats (39.1% ± 3.1%) was significantly reduced by C+P treatment (23.1% ± 5.5%) (# *p* < 0.05), while DMSO alone did not induce any neuroprotection (37.1% ± 4.4%). MCA, middle cerebral artery, C+P, chlorpromazine and promethazine.
