# Supplementary figures and images for: Reduced Apoptotic Injury by Phenothiazine in Ischemic Stroke through the NOX-Akt/PKC Pathway
Source: Brain Sci. 2019 Dec 15;9(12):378. doi: 10.3390/brainsci9120378 (PMC6955743; doi:10.3390/brainsci9120378)

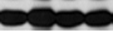

Supplement: Supplementary file 1 [file brainsci-09-00378-s001.zip › full Western blot images/BAX/Actin 24h BAX.tif]

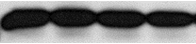

Supplement: Supplementary file 1 [file brainsci-09-00378-s001.zip › full Western blot images/BAX/Actin 6h BAX.tif]

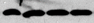

Supplement: Supplementary file 1 [file brainsci-09-00378-s001.zip › full Western blot images/BAX/BAX 24h.tif]

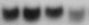

Supplement: Supplementary file 1 [file brainsci-09-00378-s001.zip › full Western blot images/BAX/BAX 6h.tif]

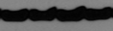

Supplement: Supplementary file 1 [file brainsci-09-00378-s001.zip › full Western blot images/Bcl-xL/Actin 24h Bcl-xL.tif]

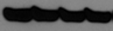

Supplement: Supplementary file 1 [file brainsci-09-00378-s001.zip › full Western blot images/Bcl-xL/Actin 6h Bcl-xL.tif]

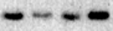

Supplement: Supplementary file 1 [file brainsci-09-00378-s001.zip › full Western blot images/Bcl-xL/Bcl-xL 24h.tif]

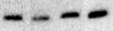

Supplement: Supplementary file 1 [file brainsci-09-00378-s001.zip › full Western blot images/Bcl-xL/Bcl-xL 6h.tif]

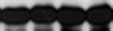

Supplement: Supplementary file 1 [file brainsci-09-00378-s001.zip › full Western blot images/Cas-3/Actin 24h Cas-3.tif]

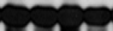

Supplement: Supplementary file 1 [file brainsci-09-00378-s001.zip › full Western blot images/Cas-3/Actin 6h Cas-3.tif]

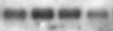

Supplement: Supplementary file 1 [file brainsci-09-00378-s001.zip › full Western blot images/Cas-3/caspase-3 24h.tif]

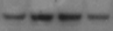

Supplement: Supplementary file 1 [file brainsci-09-00378-s001.zip › full Western blot images/Cas-3/caspase-3 6h.tif]

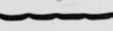

Supplement: Supplementary file 1 [file brainsci-09-00378-s001.zip › full Western blot images/P-AKT/Actin 24h p-Akt.tif]

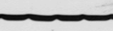

Supplement: Supplementary file 1 [file brainsci-09-00378-s001.zip › full Western blot images/P-AKT/Actin 6h p-Akt.tif]

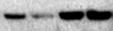

Supplement: Supplementary file 1 [file brainsci-09-00378-s001.zip › full Western blot images/P-AKT/P-Akt 24h.tif]

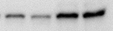

Supplement: Supplementary file 1 [file brainsci-09-00378-s001.zip › full Western blot images/P-AKT/P-Akt 6h.tif]

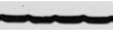

Supplement: Supplementary file 1 [file brainsci-09-00378-s001.zip › full Western blot images/PKC/Actin 24h PKC-delta.tif]

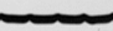

Supplement: Supplementary file 1 [file brainsci-09-00378-s001.zip › full Western blot images/PKC/Actin 6h PKC-delta.tif]

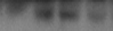

Supplement: Supplementary file 1 [file brainsci-09-00378-s001.zip › full Western blot images/PKC/PKC-delta 24h.tif]

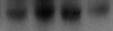

Supplement: Supplementary file 1 [file brainsci-09-00378-s001.zip › full Western blot images/PKC/PKC-delta 6h.tif]

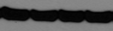

Supplement: Supplementary file 1 [file brainsci-09-00378-s001.zip › full Western blot images/cleaved Cas-3/Actin 24h cleaved Cas-3.tif]

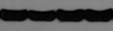

Supplement: Supplementary file 1 [file brainsci-09-00378-s001.zip › full Western blot images/cleaved Cas-3/Actin 6h cleaved Cas-3.tif]

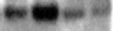

Supplement: Supplementary file 1 [file brainsci-09-00378-s001.zip › full Western blot images/cleaved Cas-3/cleaved caspase-3 24h.tif]

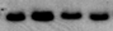

Supplement: Supplementary file 1 [file brainsci-09-00378-s001.zip › full Western blot images/cleaved Cas-3/cleaved caspase-3 6h.tif]
